# The Antiviral Spectra of TRIM5α Orthologues and Human TRIM Family Proteins against Lentiviral Production

**DOI:** 10.1371/journal.pone.0016121

**Published:** 2011-01-14

**Authors:** Seiga Ohmine, Ryuta Sakuma, Toshie Sakuma, Tayaramma Thatava, Hiroaki Takeuchi, Yasuhiro Ikeda

**Affiliations:** 1 Department of Molecular Medicine, Mayo Clinic, Rochester, Minnesota, United States of America; 2 International Research Center for Infectious Diseases, Institute of Medical Science, University of Tokyo, Tokyo, Japan; University of Minnesota, United States of America

## Abstract

**Background:**

Rhesus monkey TRIM5α (TRIM5αrh) recognizes the incoming HIV-1 core through its C-terminal B30.2(PRYSPRY) domain and promotes its premature disassembly or degradation before reverse transcription. Previously, we have shown that TRIM5αrh blocks HIV-1 production through the N-terminal RBCC domain by the recognition of Gag polyproteins. Although all TRIM family proteins have RBCC domains, it remains elusive whether they possess similar late-restriction activities.

**Methodology/Principal Findings:**

We examined the antiviral spectra of TRIM5α orthologues and human TRIM family members which have a genetic locus proximal to human TRIM5α (TRIM5αhu), against primate lentiviral production. When HIV-1 virus-like particles (VLPs) were generated in the presence of TRIM5α proteins, rhesus, African green and cynomolgus monkey TRIM5α (TRIM5αag and TRIM5αcy), but not TRIM5αhu, were efficiently incorporated into VLPs, suggesting an interaction between HIV-1 Gag and TRIM5α proteins. TRIM5αrh potently restricted the viral production of HIV-1 groups M and O and HIV-2, but not simian lentiviruses including SIV_MAC_1A11, SIV_AGM_Tan-1 or SIV_AGM_SAB-1. TRIM5αhu did not show notable late restriction activities against these lentiviruses. TRIM5αag and TRIM5αcy showed intermediate restriction phenotypes against HIV-1 and HIV-2, but showed no restriction activity against SIV production. A series of chimeric TRIM5α constructs indicated that the N-terminal region of TRIM5αag and TRIM5αcy are essential for the late restriction activity, while the C-terminal region of TRIM5αcy negatively regulates the late restriction activity against HIV-1. When select human TRIM family proteins were examined, TRIM21 and 22 were efficiently incorporated into HIV-1 VLPs, while only TRIM22 reduced HIV-1 titers up to 5-fold. The antiviral activities and encapsidation efficiencies did not correlate with their relative expression levels in the producer cells.

**Conclusions/Significance:**

Our results demonstrated the variations in the late restriction activities among closely related TRIM5α orthologues and a subset of human TRIM family proteins, providing further insights into the late restriction activities of TRIM proteins.

## Introduction

Approximately 8% of the human genome is comprised of retroviral elements, implicating an extensive history of competition between hosts and retroviruses [1], [2]. To counteract these viruses, primates have developed defensive measures which target various aspects of the retroviral life cycle. Cellular restriction factor TRIM5α is one such contributing element in this antiviral defense against retroviruses [3], [4], [5], [6]. TRIM5α belongs to the TRIM family of proteins, which are characterized by sequential domains in the N-terminal half of the protein, RING, with one or two b-boxes followed by a coiled-coil motif and its α isoform includes a C-terminal B30.2(PRYSPRY) domain. The rhesus monkey TRIM5α (TRIM5αrh) recognizes the incoming HIV-1 core through its C-terminal B30.2(PRYSPRY) domain and promotes its premature disassembly or degradation before reverse transcription [7], [8], [9], [10]. Primate TRIM5α orthologues have distinct post-entry restriction activities against a range of retro- and lentiviruses; however, they generally lack strong restriction activity against their own host-specific viruses. For instance, human TRIM5α (TRIM5αhu) restricts N-tropic murine leukemia virus (N-MLV) as well as equine infectious anemia virus (EIAV), but not human immunodeficiency virus type-1 (HIV-1) or simian immunodeficiency virus (SIV) [3], [4], [6], [11]. In contrast, TRIM5αrh expression in HIV-1-permissive cells confers strong antiviral activity against HIV-1, EIAV, N-MLV and SIV from African green monkeys (SIV_AGM_), but not against SIV from rhesus macaques (SIV_MAC_) [3], [4], [6], [9], [11], [12]. The African green monkey TRIM5α orthologue (TRIM5αag) restricts HIV-1, SIV_MAC_, EIAV and N-MLV, but not SIV_AGM_ [3], [11], while the cynomolgus monkey orthologue (TRIM5αcy) restricts HIV-1 and HIV-2, but not SIV_MAC_ infection [13]. These post-entry restriction patterns of TRIM5α orthologues suggest that lentiviruses have evolved to evade TRIM5α-mediated post-entry restriction when colonizing respective species. In response, host species also appear to have evolved their TRIM5α proteins, especially the coiled-coil and B30.2(PRYSPRY) domains, against retro- and lentiviruses [14], [15].

TRIM5αrh also exhibits an additional antiviral activity against HIV-1 production, independently of the well-characterized post-entry restriction, to block the late phase of HIV-1 replication [16], [17]. High levels of TRIM5αrh expression blocks HIV-1 production predominantly by reducing the number of HIV-1 virions, while modest TRIM5αrh expression blocks the late phase of HIV-1 replication by reducing virion infectivity as well as virion numbers [16], [18]. When HIV-1 virus-like-particles (VLPs) are produced in the presence of TRIM5αrh, TRIM5αrh is efficiently incorporated into VLPs, implicating the interaction between cellular and viral components during viral assembly [16]. This TRIM5αrh-mediated restriction of HIV-1 production is mediated by the N-terminal RBCC domain, but not the C-terminal B30.2(PRYSPRY) domain [16]. Further studies have identified several determinants for this late restriction. A RING structure is essential for the efficient interaction with HIV-1 Gag, while two amino acid residues in TRIM5αrh coiled-coil domain (M133 and T146) are critical for the late restriction activity [19]. Our data suggest that the TRIM5αrh-mediated late restriction involves at least two distinct activities: (i) interaction with HIV-1 Gag polyprotein through the N-terminal, RING and b-box 2 regions of a TRIM5αrh monomer, and (ii) an effector function(s) that depends upon the coiled-coil and linker 2 domains of TRIM5αrh [19]. Although TRIM5αhu does not show strong late restriction activities against HIV-1 group M viruses [16], it remains to be determined if the human orthologue has any late restriction activity against other human and non-human primate lentiviruses.

Previous studies have shown that TRIM5α is interferon-responsive [20], [21]. Recent study identified TRIM6, 21, 22 and 34, which are located on chromosome 11p together with TRIM5, are also interferon responsive [22]. Given that the late restriction of TRIM5α is dependent on the RBCC domain and these TRIM family proteins possess an N-terminal RBCC, it is possible that TRIM5αhu paralogues may have similar late-restriction activities. In the present study, we determined the antiviral spectra and encapsidation efficiency of TRIM5α orthologues and paralogues. We found that TRIM5α orthologues from African green (TRIM5αag) and cynomolgus (TRIM5αcy) monkeys have similar, but weaker late restriction activities against HIV-1 production. Similar to TRIM5αrh, TRIM5αag and TRIM5αcy proteins were efficiently incorporated in HIV-1 VLPs, and their RBCC domains were essential for the late restriction activities. Intriguingly, the C-terminal regions of TRIM5αag and TRIM5αcy proteins negatively regulated the late restriction activities. Studies using human TRIM5 paralogues with conserved RBCC domains demonstrated that TRIM21 and TRIM22 specifically incorporated into HIV-1 VLPs, while only TRIM22 mildly restricted HIV-1 production. Our results therefore demonstrate the variable late restriction activities of TRIM5α orthologues and paralogues. The involvement of the C-terminal sequences of TRIM5α proteins in determining the potency of late restriction activities suggests more complex mechanisms underlying TRIM protein-mediated late restriction activities than previously reported.

## Methods

### Cell culture

293T and GHOST(3)R3/X4/R5 [23] cells were maintained in Dulbecco's Modified Eagle's Medium with 4.5 g/L glucose, supplemented with 10% fetal bovine serum (FBS) and antibiotics.

### Plasmids

TRIM5αrh- or TRIM5αhu-expressing plasmids with a C-terminal HA tag, pRhT5α and pHuT5α, respectively, and codon-optimized HIV-1 GagPol expression construct pH-G/P were described previously [16]. pcDNA3.1-based TRIM5αag expression plasmid (pAgmT5α) was generated from pDON-aT5 [18] and TRIM5αcy cDNA was amplified from total RNA isolated from cynomolgus monkey lymphoid line, HSC-F cells (kindly provided by Dr. Hirofumi Akari) and cloned into pcDNA3.1 to generate pCynT5α. Proviral lentivirus plasmids pNL4-3, p89.6, p94UG114.1, pSIVmac1A11, pSIVagmTan-1 and pSAB-1 were obtained from the NIH AIDS Research and Reference Reagent Program [24], [25], [26], [27], [28], [29], [30], [31]. pROD10 was described previously [32]. pCMO2.41 and pCMO2.5 were generously provided by Dr. Hans-Georg Kräusslich. Chimeric TRIM5α-expression plasmids were generated using the conserved *Hind*III sites in the coiled coil domain of TRIM5α sequences, and cloned into pcDNA3.1. N-terminally tagged TRIM1, 6, 18, 21, 22 and 34 were kindly provided by Dr. Paul Bieniasz [33]. Since modifications in the N-terminal region of TRIM5αrh ablated its late restriction activity [16], we generated C-terminally HA-tagged human TRIM expression plasmids based on pcDNA3.1(+) (Invitrogen) and verified their sequences. TRIM5αrh point mutants were generated using the QuikChange II XL site-directed mutagenesis kit (Stratagene, Cedar Creek, TX).

### Confocal microscopy analysis

293T cells were transfected with 1.0 µg TRIM5α-expression plasmid using FuGene 6 (Roche, Madison, WI) in a 6-well culture plate. 6-h post-transfection, 293T cells were seeded into LabTek II 8-well chamber slides (Nunc, Rochester, NY) at approximately 1.5×10^5^ cells/ml. 36 h post-transfection, cells were fixed in 4% paraformaldehyde, permeabilized on ice using 0.1% saponin after fixation then blocked with 5% FBS PBS solution for 30 min at room temperature. Primary antibody (rat anti-HA, 1∶250, Roche) and secondary antibody (FITC-conjugated goat anti-rat IgG, 1∶250, Thermo Scientific, Waltham, MA) were used to visualize HA signals. Images were obtained using the Zeiss LSM 510 confocal microscope (Carl Zeiss MicroImaging, Inc., Thornwood, NY) and analyzed with the Zeiss LSM Image Browser software.

### Western Blotting

Proteins were subjected to SDS-PAGE in a 4–15% Tris-HCl gel and then transferred onto a PVDF membrane at 0.7 mA/cm^2^ for 40 min. Membranes were blocked in 5% milk PBS overnight prior to application of antibodies. Antibodies were used in the following concentrations: rat anti-HA (1∶1000, Roche), and mouse anti-β-actin (1∶1000). HIV-1 Gag proteins were detected using a mixture of anti-p24 antibodies (183-H12-5C, 1∶1000 and AG3.0, 1∶500) [34], [35]. Peroxidase-conjugated secondary antibodies (goat anti-rat IgG and goat anti-mouse IgG, Thermo Scientific) were used at a 1∶2000 concentration.

### Virus-like-particle incorporation assay

1.0 µg of TRIM5α- or TRIM-expressing plasmid and 0.2 µg of a codon-optimized HIV-1 Gag-Pol expression plasmid, pH-GP [36], were co-transfected into 293T cells using FuGene 6. Two days post-transfection, transfected cells were harvested in RIPA buffer to assess TRIM5α and HIV-1 GagPol expression. Culture supernatants were also harvested, and passed through a 0.45 µm-pore syringe filter for VLP purification. The filtered supernatants were then centrifuged at 18,000×g for 90 min through a 20% sucrose cushion, resuspended in PBS and centrifuged at 18,000×g for 90 min. Pelleted VLPs were lysed in 5 µl of RIPA buffer and to which 5 µl of sample buffer was added, heat-denatured and subjected to immunoblot analysis.

### Viral production assay

For the assessment of TRIM5α orthologue antiviral activities, increasing amounts of pHuT5α, pRhT5α, pAgmT5α and pCynT5α (0.1, 0.3 and 1.0 µg) were co-transfected with 0.1 µg of infectious lentiviral proviral plasmid into 293T cells (1.0×10^6^ cells) using FuGene 6 (Roche). A control plasmid (pBlueScriptIIKS(+), Stratagene) was added to each transfection reaction to bring the final plasmid concentrations to 1.2 µg per transfection. Human TRIM family protein antiviral activities were examined similarly, except 1.0 µg of TRIM-expression plasmid was used in each reaction. Two days post-transfection, cellular supernatants were passed through a 0.45 µm-pore syringe filter and the viral titers in the supernatants were determined as infectious units/ml (IU/ml) in GHOST(3)R3/X4/R5 indicator cells.

## Results

### Late restriction activities of TRIM5αrh, TRIM5αag and TRIM5αcy against primate lentiviruses

**Figure 1:**
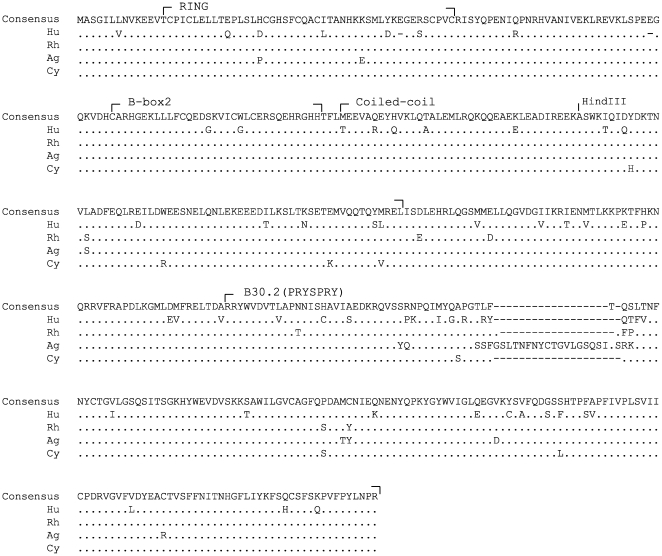
Amino acid sequence alignment of TRIM5α orthologues examined in this study. Consensus amino acid sequences of TRIM5α. as well as TRIM5αhu (Hu), TRIM5αrh (Rh), TRIM5αag (Ag) and TRIM5αcy (Cy) amino acid sequences are shown. Identical residues are indicated by dots. Gaps are indicated by dashes. HindIII site in the coiled-coil domain was used as the junction for the chimeras used in this study.

In the well-characterized TRIM5α post-entry restriction, the TRIM5α B30.2(PRYSPRY) determines the potency and specificity of restriction. In contrast, the late restriction of HIV-1 depends on the TRIM5α RBCC domain [16], [19]. The highly conserved RBCC sequences among simian TRIM5α orthologues suggest the possible late restriction activities in other primate TRIM5α proteins (Fig. 1). We therefore examined the late-restriction activities of TRIM5αag and TRIM5αcy against a series of human and non-human primate lentiviruses. Human lentiviruses, HIV-1 group M viruses, HIV-1 group O and HIV-2 viruses were examined for their sensitivity to TRIM5α-mediated late restriction. HIV-1 group O viruses are divergent from group M viruses [37] and show different sensitivities to TRIM5αrh-mediated Lv1 restriction [38], partly due to their cyclophilin A-independence [38], [39]. Since group O and HIV-2 viruses have been less successful in colonizing human population than the more-prevalent HIV-1 group M viruses, we hypothesized that TRIM5αhu might be able to block the late phase of group O HIV-1 or HIV-2 replication. SIV_MAC_ and SIV_AGM_ were also included to test the late restriction activities of TRIM5α proteins against non-human primate lentiviruses.

**Figure 2:**
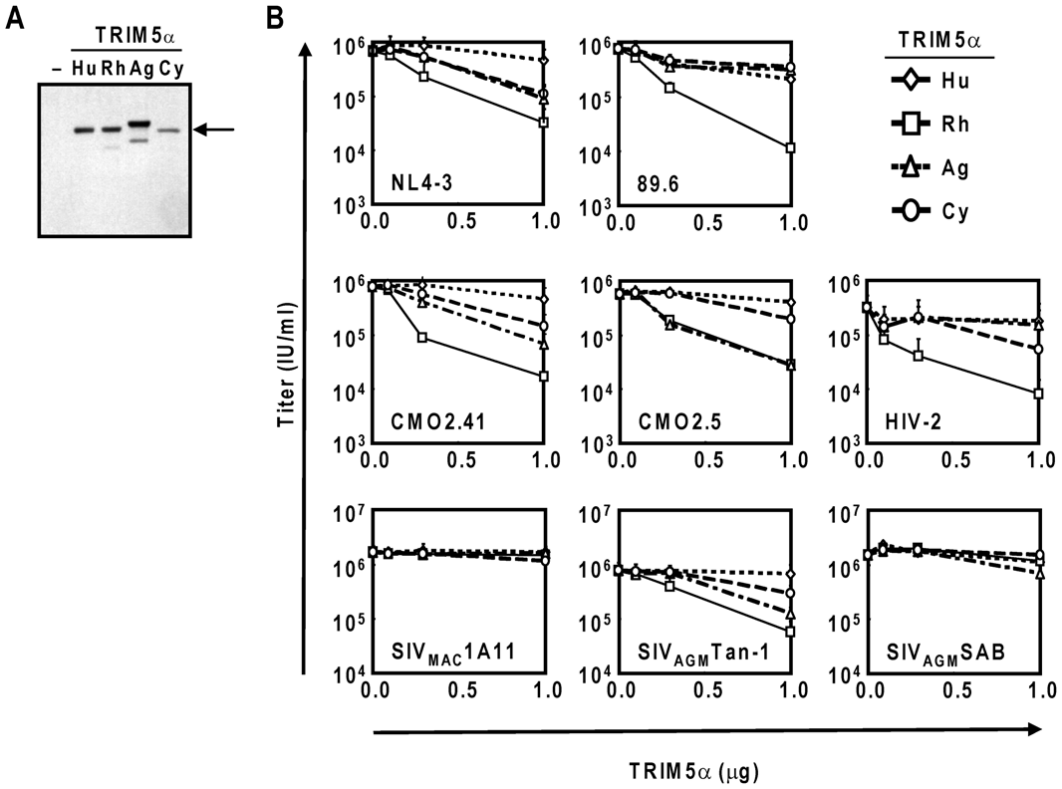
TRIM5α orthologue expression in 293T cells and antiviral spectrum of TRIM5α orthologues against lentiviral production. (A) Verification of the proper expression of TRIM5α orthologues. Control plasmid (−), TRIM5αhu (Hu), TRIM5αrh (Rh), TRIM5αag (Ag) and TRIM5αcy (Cy) expression in transfected 293T cells were verified via immunoblot analysis. Arrow depicts approximate TRIM5α band size. (B) 293T cells were co-transfected with a primate lentivirus proviral plasmid and increasing amounts of human (Hu), rhesus monkey (Rh), African green monkey (Ag) or cynomolgus monkey (Cy) TRIM5α-expressing plasmids. As infectious proviral plasmids, HIV-1 Group M (pNL4-3 and p89.6), HIV-1 Group O (pCMO2.41, pCMO2.5), HIV-2 (pROD10), and SIV (pSIV_MAC_1A11, pSIV_AGM_Tan-1 and pSIV_AGM_SAB-1) were used. Viral titers were determined in GHOST(3)R3X4R5 indicator cells and described as infectious units per ml (IU/ml). Error bars indicate one standard deviation.

Immunoblot analysis was performed to verify the proper expression of TRIM5αag and TRIM5αcy proteins following transfection into 293T cells (Fig. 2A). TRIM5αrh reduced the titers of two HIV-1 group M clones (NL4-3, 89.6) and HIV-1 group O clones (CMO2.41 and CMO2.5) by up to 60-fold in a dose-dependent manner (Fig. 2B). TRIM5αrh also reduced the titers of HIV-2 up to 40-fold. Although SIV_MAC_ and SIV_AGM_SAB-1 titers were largely unaffected by TRIM5αrh, we observed a 10-fold decrease in the titers of SIV_AGM_Tan-1 in the presence of TRIM5αrh (Fig. 2B). TRIM5αag and TRIM5αcy showed similar patterns of late restriction activity to its rhesus monkey orthologue, although the reductions in viral titers were modest. NL4-3 titers were reduced by 7-fold in cells expressing TRIM5αag or TRIM5αcy, while 89.6 titers were only reduced by 2-fold. Although group O isolate CMO2.41 virus titers were reduced by up to 7-fold when producer cells expressed TRIM5αag or TRIM5αcy, the titers of another Group O clone CMO2.5, which is based on the CMO2.41 isolate but containing the 5′LTR to *vpr* sequences from the MVP2171 O-type isolate [40], were strongly affected by TRIM5αag but not TRIM5αcy. TRIM5αag and TRIM5αcy showed little late restriction activity on SIV_MAC_1A11, SIV_AGM_Tan-1, SIV_AGM_SAB-1 and HIV-2 production. Intriguingly, TRIM5αag reduced SIV_AGM_Tan-1 titers by 6-fold (Fig. 2B), which may be explained by the difference in the host species of SIV_AGM_ and TRIM5αag: SIV_AGM_Tan-1 is isolated from the *Chlorocebus tantalus* [31] but the TRIM5αag protein used in these experiments were derived from *Chlorocebus aethiops*-derived CV1 cells [41]. TRIM5αhu marginally reduced HIV titers, while it did not affect SIV titers (Fig. 2B).

### Efficient encapsidation of rhesus monkey, African green monkey and cynomolgus monkey TRIM5α into HIV-1 virus-like particles

**Figure 3:**
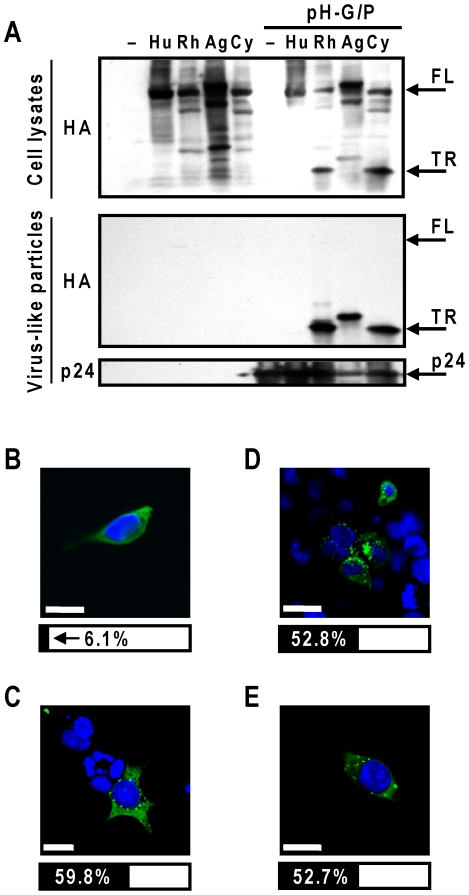
TRIM5α orthologue incorporation into HIV-1 virus-like particles and subcellular localization in 293T cells. (A) HIV-1 VLP encapsidation assays using human and simian TRIM5α orthologues. 293T cells were co-transfected with 1.0 µg of TRIM5α-expressing plasmid and 0.2 µg of a codon-optimized HIV-1 Gag-Pol expression plasmid (pH-GP). Producer cell proteins and supernatant containing VLPs were harvested two days post-transfection. Upper panel shows immunoblot analysis of producer cell lysates probed against TRIM5α (anti-HA). Lower two panels shows immunoblot analysis of VLPs probed against TRIM (anti-HA), HIV-1 (anti-p24; 183-5C-H12, 1∶1000 and AG3.0, 1∶500). Full length (FL), truncated (TR) TRIM5α bands and HIV-1 CA (p24) are indicated by the arrows. (B–E) 293T cells were transfected with TRIM5α-expressing plasmid and seeded onto 8-well chamber slides 6h post-transfection. Cells were fixed with paraformaldehyde and permeabilized with 0.1% saponin, then stained against TRIM5αHA tag using rat anti-HA antibody 3F10 and a FITC conjugated anti-rat IgG secondary antibody. Representative TRIM5αhu (B), TRIM5αrh (C), TRIM5αag (D) and TRIM5αcy (E) images are shown. Bar under the respective images represent the percentages of cells exhibiting more than three discrete cytoplasmic bodies (black) or a diffuse signal void of cytoplasmic bodies (white). Scale bars represent 20 µm.

Previously, we have shown that when HIV-1 proteins were produced in the presence of TRIM5αrh, HIV-1 Gag polyproteins were rapidly degraded [16]. In contrast, over-expression of codon-optimized HIV-1 GagPol was able to saturate the late restriction activity, leading to production of sufficient amounts of VLPs in the presence of TRIM5αrh and efficient incorporation of TRIM5αrh in the VLPs [16], [17]. Efficient encapsidation of TRIM5αrh into VLPs generated without HIV-1 protease suggests specific interaction between TRIM5αrh and HIV-1 Gag before or during HIV-1 assembly [16]. HIV-1 protease appears to cleave TRIM5αrh in the B30.2(PRYSPRY) domain to produce the truncated, 20 kDa form of TRIM5αrh in the VLPs, because formation of the 20 kDa form was not seen in the VLPs made without HIV-1 Pol, or in the VLPs treated with HIV-1 protease inhibitors [16]. TRIM5αrh and TRIM5αhu chimeric constructs demonstrated that processing of TRIM5α proteins was not necessary for their late restriction activities [16], [17]. Since three simian TRIM5α proteins showed different late restriction activities (Fig. 2B), we examined the differences in their HIV-1 Gag-association efficiencies by the VLP encapsidation assay. When high levels of HIV-1 VLPs were generated in the presence of TRIM5α proteins, efficient incorporation of truncated forms of TRIM5αrh, TRIM5αag and TRIM5αcy, but not TRIM5αhu, were observed (Fig. 3A). The truncated forms of TRIM5α likely resulted from the cleavage of TRIM5α proteins in the B30.2(PRYSPRY) domain by the HIV-1 protease. The efficient incorporation of the simian TRIM5α proteins into VLPs suggests a specific interaction between simian TRIM5α proteins and HIV-1 Gag proteins. Since this experiment was performed under conditions where the late restriction activities were saturated by the over-expressed HIV-1 Gag, the slight reduction of p24 in the TRIM5αag lane (Fig 3A) did not reflect the level of restriction.

### Simian TRIM5α orthologues form prominent cytoplasmic bodies

TRIM5α proteins self-associate to form cytoplasmic bodies, while dimerization is also required for efficiently binding to retroviral capsid [42], [43]. Immunohistochemistry studies in 293T cells suggest that TRIM5αrh efficiently form cytoplasmic bodies, while TRIM5αhu primarily displayed more diffused cytoplasmic localizations [19]. In order to address whether the varying late restriction activities of three simian TRIM5α proteins were due to their different subcellular localizations, we determine the localizations of simian TRIM proteins by immunostaining. 293T cells were transfected with 1.0 µg of TRIM5α-expressing plasmid. TRIM5α proteins were then detected with anti-HA antibodies and analyzed by confocal microscope. TRIM5αhu, which does not strongly affect HIV-1 production, showed predominantly diffuse cytoplasmic distribution with little discernible cytoplasmic bodies (Fig. 3B). In contrast, 59.8%, 52.8% and 52.7% of 293T cells expressing TRIM5αrh, TRIM5αag and TRIM5αcy, respectively, showed discrete cytoplasmic bodies (>3 discrete cytoplasmic body formations per cell) throughout the cytoplasm (Fig. 3 C–E). Our data therefore demonstrated similar subcellular localizations of TRIM5αrh, TRIM5αag and TRIM5αcy, despite their varying late restriction activities.

### The RBCC sequences of TRIM5αag and TRIM5αcy are essential for the late restriction activity

**Figure 4:**
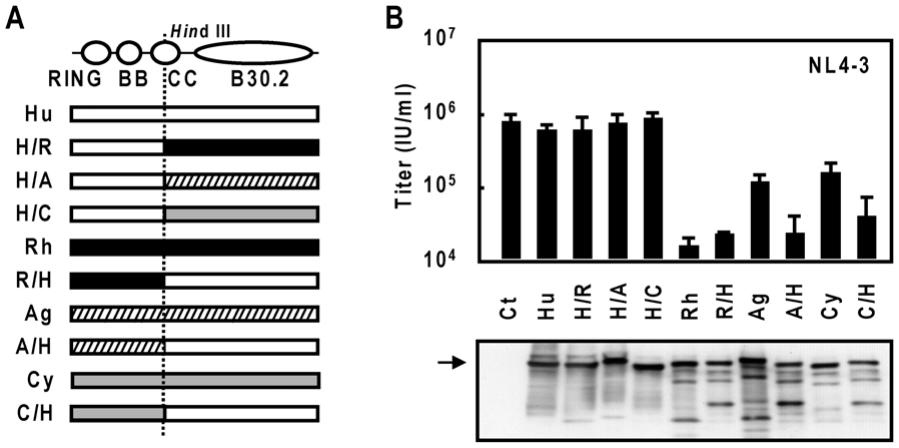
Antiviral activities of chimeric TRIM5α proteins against HIV-1 production. (A) Schematic representation of the chimeric TRIM5α constructs between human (Hu), rhesus monkey (Rh), African green monkey (Ag) and cynomolgus monkey (Cy) TRIM5α proteins. (B) Upper panel: late-restriction activities of chimeric TRIM5α proteins upon co-transfection with pNL4-3 into 293T cells. Viral titers were determined in GHOST(3)R3X4R5 indicator cells and reported as infectious units per ml (IU/ml). Error bars indicate one standard deviation. Lower panel: western blot analysis of chimeric TRIM5α proteins following transfection into 293T cells. Arrow indicates approximate full-length TRIM5α size.

TRIM5αhu amino acid sequences are the most divergent and showed the weakest late restriction activity among the tested TRIM5α orthologues (Fig. 2B). We therefore assessed whether the substitution with TRIM5αhu N- or C-terminal sequences can relieve the late restriction activities of the simian TRIM5α orthologues. Chimeric TRIM5α constructs were generated as depicted in Figure 4A. Simian TRIM5α proteins with N-terminal TRIM5αhu sequences showed no effect on HIV-1 (Fig. 4B, upper panel) or SIV_MAC_ production (data not shown), underscoring the importance of the RBCC sequences in the late restriction activity against HIV-1. Although the TRIM5αrh-TRIM5αhu chimera, R/H, showed a late restriction activity as potent as wild-type TRIM5αrh, the two simian TRIM5α chimeras with human N-terminal sequences, A/H and C/H, showed stronger late restriction activities than wild-type TRIM5αag and TRIM5αcy (Fig. 4B, upper panel). No prominent differences in protein expression levels were observed between the chimeric TRIM5α proteins (Fig. 4B, lower panel). These data indicate that the RBCC sequences of TRIM5αag and TRIM5αcy are essential for the late restriction activity and suggest the possibility that the C-terminal regions of simian TRIM5αag and TRIM5αcy proteins negatively regulate TRIM5α late restriction activities against HIV-1.

### The influence of variations in the RBCC sequences of simian TRIM5α proteins on the late restriction activities

**Figure 5:**
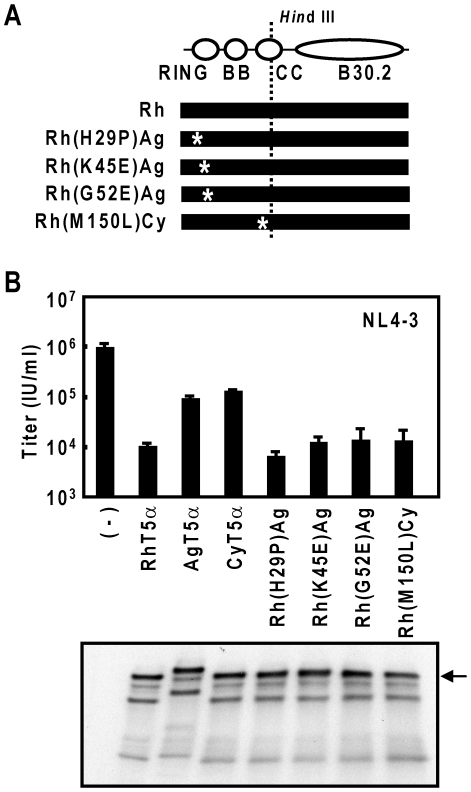
Antiviral activities of TRIM5αrh proteins with single-amino acid substitutions in the RBCC region against HIV-1. (A) Schematic representation of TRIM5αrh constructs carrying single amino acid residue substitutions. Single residue substitutions corresponding to TRIM5αag sequences were introduced into C-terminally HA-tagged TRIM5αrh (Rh) to generate Rh(H29P)Ag, Rh(K45E)Ag and Rh(G52E)Ag. 150L TRIM5αcy sequence was introduced into TRIM5αrh to generate Rh(M150L)Cy. (B) Upper panel: viral titers of HIV-1 NL4-3 generated in the presence of TRIM5α proteins carrying single amino acid substitutions. Viral production in the presence of TRIM5αrh (RhT5α), TRIM5αag (AgT5α) and TRIM5αcy (CyT5α) are shown for comparison. Viral titers were determined in GHOST(3)R3X4R5 indicator cells and reported as infectious units per ml (IU/ml). Error bars indicate one standard deviation. Lower panel: western blot analysis of single-residue-substituted TRIM5αrh proteins following transfection into 293T cells. Arrow indicates approximate full-length TRIM5α size.

Previously, we demonstrated that introduction of two TRIM5αhu-specific amino acid residues into TRIM5αrh (M133T and T146A in the coiled-coil region) abrogates the late restriction activity of TRIM5αrh [19]. In the RING, b-box 2 and partial coiled-coil domains, there are three amino acid differences between TRIM5αrh and TRIM5αag, while only one residue separates TRIM5αrh and TRIM5αcy. In order to address the possible contributions of these residues on the modest late restriction activities of TRIM5αag and TRIM5αcy, we introduced single amino-acid substitutions into the N-terminal half of TRIM5αrh using corresponding TRIM5αag or TRIM5αcy sequences (Fig. 5A). After verification of similar chimeric TRIM5α expression levels by immunoblot (Fig. 5B, lower panel), we examined respective late restriction activities against HIV-1. When HIV-1 NL4-3 was produced in the presence of TRIM5αrh, TRIM5αag and TRIM5αcy, HIV-1 titers were reduced approximately 60-, 7- and 6-folds, respectively, while the TRIM5αrh mutants with single amino acid substitutions, Rh(H29P)Ag, Rh(K45E)Ag, Rh(G52E)Ag and Rh(M150L)Cy, showed potent late restriction activities, comparable to that of wild-type TRIM5αrh (Fig. 5B, upper panel). These data indicate that variations in the single amino acid residues alone (29P, 45E, 52E or 150L) cannot explain the modest late restriction activities of TRIM5αcy and TRIM5αag.

### C-terminal sequences of TRIM5αag and TRIM5αcy negatively regulates the potency of late restriction activity

**Figure 6:**
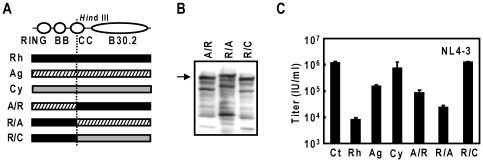
The effects of African green and cynomolgus monkey TRIM5α C-terminal sequences on TRIM5αrh-mediated HIV-1 late restriction activities. (A) Schematic representation of the chimeric TRIM5α constructs between rhesus monkey (Rh, filled), African green monkey (Ag, hatched) and cynomolgus (Cy, dotted) TRIM5α proteins. (B) Western blot analysis of chimeric TRIM5α proteins A/R, R/A and R/C following transfection into 293T cells. Arrow indicates approximate full-length TRIM5α size. (C) Late-restriction activities of chimeric TRIM5α proteins upon co-transfection with pNL4-3 into 293T cells. Viral titers were determined in GHOST(3)R3X4R5 indicator cells and reported as infectious units per ml (IU/ml). Error bars indicate one standard deviation.

To test whether TRIM5αag or TRIM5αcy C-terminal sequences can impair the late restriction activity against HIV-1, we generated chimeric TRIM5α constructs as depicted in Figure 6A. Similar levels of TRIM5α expression were confirmed via immunoblot (Fig. 6B). Although the restriction activity of the TRIM5αrh protein with N-terminal TRIM5αcy sequence (RhM150LCy) did not notably differ from wild-type TRIM5αrh restriction activities (Fig. 4B), the restriction against HIV-1 was relieved when C-terminal TRIM5αcy sequences were fused with the N-terminal region of TRIM5αrh (Fig. 6C). TRIM5αag C-terminal sequences in TRIM5αrh (R/A) impaired the late restriction against HIV-1 by 3-fold when compared to wild-type TRIM5αrh (Fig. 6C). These data suggest that C-terminal sequences of TRIM5α proteins can negatively regulate the late restriction activities, offering partial explanation to the modest late restriction activities of TRIM5αag and TRIM5αcy against HIV-1.

### Encapsidation and late restriction activities of human TRIM proteins

The 5α isoform of human TRIM protein had very little effect on HIV-1 production; however TRIM5αhu is one of over 80 members of the RBCC family of TRIM proteins. We therefore sought to examine whether other human TRIM family proteins may (1) be incorporated into HIV-1 VLPs and (2) restrict HIV-1 production. From the vast numbers of TRIM family proteins, we examined TRIM6, TRIM34 and TRIM22 since they are located in a paralogous cluster which includes TRIM5α [44]. We also examined the influence of TRIM1, TRIM18 and TRIM21 expression on HIV-1 VLP incorporation. TRIM1 has antiviral activity against N-tropic murine leukemia virus infection [6] and TRIM18 is its paralogue. TRIM21 can modulate TRIM5α ubiquitination [45] as well as the interferon-mediated antiviral response [46].

**Figure 7:**
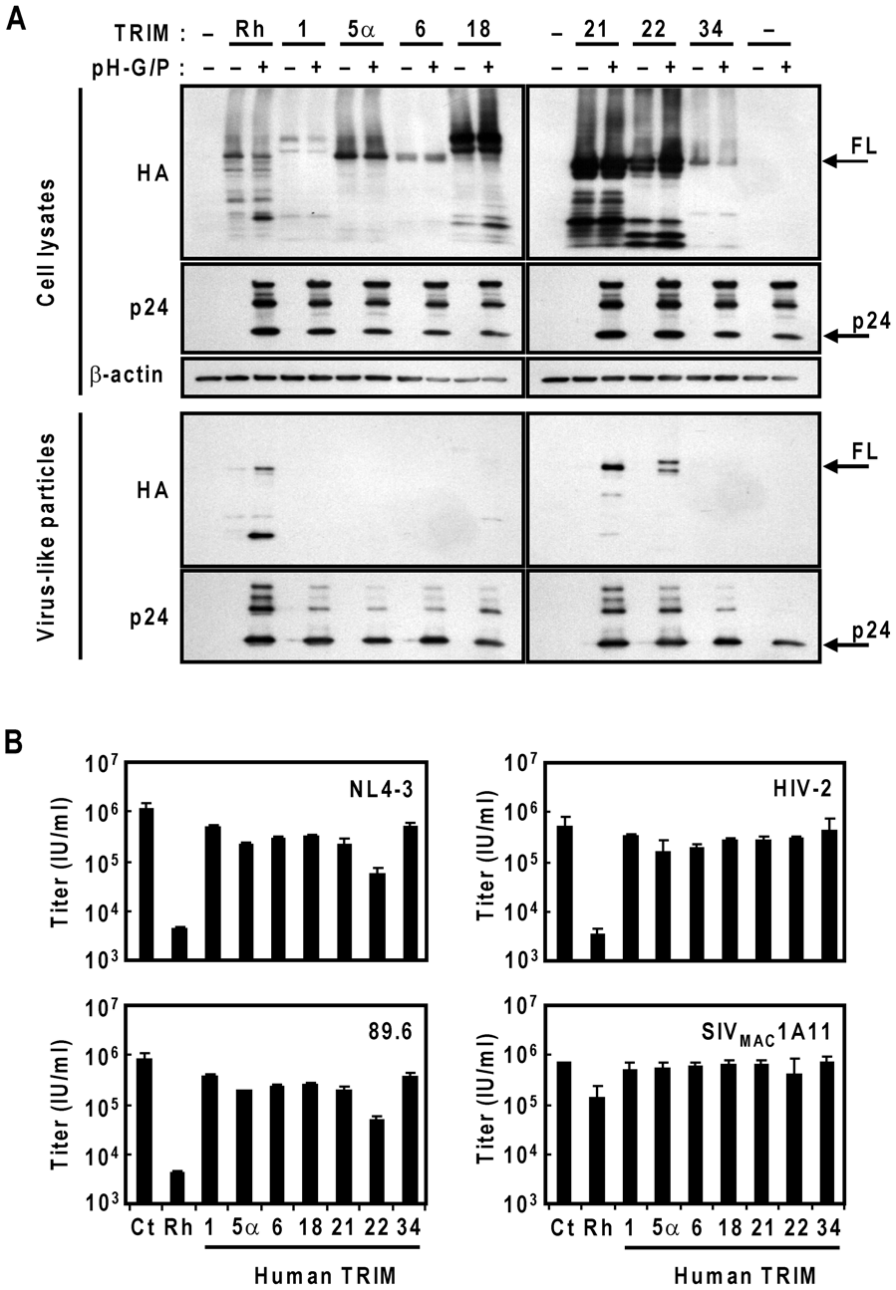
HIV-1 VLP encapsidation and late restriction activities of human TRIM proteins. (A) Immunoblot analyses of C-terminally HA-tagged human TRIM proteins (1, 5α, 6, 18, 21, 22 and 34) with or without codon-optimized HIV-1 Gag-Pol expression plasmid (pH-G/P) are shown. TRIM5αrh was used as a positive control for incorporation. Top three panels show proteins harvested from producer cell lysates, immunoblotted against TRIM (anti-HA), HIV-1 (anti-p24; 183-5C-H12, 1∶1000 and AG3.0, 1∶500) or actin (anti-β-actin), while the bottom two panels show immunoblots of VLPs harvested from respective producer cells against TRIM (anti-HA) or HIV-1 (anti-p24; 183-5C-H12, 1∶1000 and AG3.0, 1∶500). FL designates approximate full-length TRIM size, and p24 designates HIV-1 p24 capsid size. (B) 293T cells were co-transfected with 1.0 µg of TRIM-expressing plasmid: TRIM5αrh, TRIM1, TRIM5αhu, TRIM6, TRIM18, TRIM21, TRIM22 and TRIM34, with 0.1 µg of lentivirus proviral plasmid: pNL4-3, p89.6, pROD10 or pSIV_MAC_1A11. Viral titers were determined in GHOST(3)R3X4R5 indicator cells and reported as infectious units per ml (IU/ml). Error bars indicate one standard deviation.

To obtain sufficient amounts of VLPs in the presence of TRIM proteins, we used pH-GP, which generates high levels of HIV-1 GagPol and abrogates the late restriction activities of TRIM5αrh [16]. 293T cells were co-transfected with 1.0 µg of TRIM-expressing plasmids and 0.25 µg of codon-optimized HIV-1 GagPol-expression plasmid, pH-GP. Cell lysates and HIV-1 VLPs were harvested as described in materials and methods. Immunoblot analysis was performed to detect HA-tagged TRIM proteins as well as HIV-1 Gag proteins. All TRIM proteins were detected in the producer cells (Fig. 7A). Efficient VLP incorporation was evident with TRIM5αrh, human TRIM21 and TRIM22 (Fig. 7A). Previously, we have demonstrated that HIV-1 Gag maturation delays in the presence of TRIM5α, resulting in accumulation of premature Gag proteins in producer cells and VLPs [16], [17]. This was also true with human TRIM21 and TRIM22, where VLPs made in the presence of TRIM21 and TRIM22 showed notable accumulation of premature Gag proteins, particularly in VLPs (Fig. 7A). No HA signal was detected in the VLPs made in the presence of TRIM1, 6 and 34 (Fig. 7A). Intracellular expression levels of the TRIM proteins were not necessarily correlated with their incorporation into VLPs, indicating that the incorporation of TRIM proteins is not due to non-specific packaging of RBCC proteins into VLPs. These observations suggest the direct or indirect interaction of TRIM21 and TRIM22 with HIV-1 Gag proteins in producer cells. No effects on HIV-1 Gag levels in the presence of TRIM5α or TRIM22 are likely due to the saturation of late restriction activities by over-expression of codon optimized HIV-1 Gag.

Next, we examined the correlation between VLP incorporation status of human TRIM proteins and late restriction activities. To assess the antiviral activities of these TRIM proteins, 1.0 µg of TRIM-expressing plasmids were co-transfected with 0.1 µg of pNL4-3, p89.6, pROD10 or pSIVmac1A11 into 293T cells, and viral titers were determined in GHOST indicator cells. Of the human TRIM proteins that were assessed, only TRIM22 showed a slight reduction in NL4-3 and 89.6 titers, while the other TRIM proteins had no effect (Fig. 7B). None of the human TRIM proteins showed notable effects on HIV-2 or SIV_MAC_1A11 production (Fig. 7B).

## Discussion

In this report, we examined the late restriction activities and VLP encapsidation efficiencies of simian TRIM5α orthologues and related human TRIM proteins, and their late restriction activities against a panel of lentiviruses. Our results revealed the antiviral spectra and varying restriction activities of these TRIM proteins against lentiviral production. The relative expression levels or subcellular localizations of TRIM5α could not explain the encapsidation efficiency or the potency of late restriction activity. The RBCC domains of TRIM5αag and TRIM5αcy were essential for the late restriction, while the C-terminal regions of TRIM5αag and TRIM5αcy negatively regulated the restriction activities. Similar antiviral spectra between simian TRIM5α orthologues may suggest a conserved restriction mechanism among these proteins. Of the examined human TRIM5 paralogues, TRIM21 and TRIM22 were efficiently incorporated into HIV-1 VLP, while only TRIM22 showed marginal late restriction activity.

Among primate lentiviruses, HIV-2 and SIV_MAC_ are closely related, as sooty mangabey SIV has transferred to humans and rhesus monkeys as HIV-2 and SIV_MAC_ [47]. However, TRIM5αrh blocks the infection of HIV-2, but not SIV_MAC_, mainly due to the difference in the structure in the capsid protein that recruits cyclophilin A into HIV-1 virions [48]. Examination of HIV-2 and SIV_MAC_ production in the presence of simian TRIM5α proteins demonstrated the most remarkable differences between the two closely related lentiviruses. Although SIV_MAC_ was resistant to all four TRIM5α orthologues in the late-restriction, up to a 40-fold reduction in HIV-2 production was observed in the presence of TRIM5αrh. It is possible that the variations in the CA loop regions of SIV_MAC_ and HIV-2, which correspond to the HIV-1 CA cyclophilin-binding loop, may in part contribute to the differential late restriction phenotypes. However, the CA loop region cannot solely explain the resistance of SIV_MAC_ to the TRIM5αrh-mediated late restriction, because an HIV-1 cyclophilin-binding loop mutant with corresponding SIV_MAC_ sequence was still sensitive to the late restriction [36]. Intriguingly, a recent study has shown that a single amino acid change in the HIV-1 CA cyclophilin-binding loop allowed the virus to escape from TRIM5αrh-mediated post-entry restriction, suggesting the importance of the CA loop structure for restriction factor recognition [49]. Our previous study has also suggested that SIV_MAC_ resists TRIM5αrh-mediated late restriction by counteracting or saturating the TRIM5α late restriction machinery, rather than escaping TRIM5αrh recognition altogether [36]. We therefore speculate that the sensitivity of HIV-2 to TRIM5αrh-mediated late restriction is partly due to the relatively inefficient HIV-2 production from pROD10, where production of progeny virions may not be sufficient to overcome the TRIM5αrh-mediated late restriction. The different replication kinetics of CMO2.41 and CMO2.5 in PBMCs can be attributed to the *gag-pol* region of CMO2.5, which is derived from a separate primary type-O isolate [50]. The differences in viral gag-pol sequences offers partial explanation as to why CMO2.41 and CMO2.5 responded differently to the late restriction activities of TRIM5αag (Fig. 2B).

The incorporation of TRIM5αrh into the VLPs made with HIV-1 Gag suggests a specific interaction between TRIM5αrh and HIV-1 Gag polyproteins [16]. Determinants for this interaction lie in the RING and coiled-coil domains of TRIM5αrh, and the B30.2(PRYSPRY) motif is not required for the interaction or the late restriction activity of TRIM5αrh [19]. TRIM5αrh mutants with the M133T and/or T146A amino acid substitutions in TRIM5αrh coiled-coil domain showed efficient encapsidation but impaired late restriction activity [19]. Although the RBCC sequences, including the M133 and T146, of three simian TRIM5α proteins are highly conserved among the three simian TRIM5α proteins, we found that TRIM5αag and TRIM5αcy showed weaker late restriction activities than TRIM5αrh. Similar to our previous study, replacement of the N-terminal RBCC sequences of TRIM5αag and TRIM5αcy with the corresponding TRIM5αhu sequences resulted in the loss of prominent late restriction effects of these two simian TRIM5α proteins, indicating the essential roles of the RBCC domains in their late restriction activities (Fig. 4B). Unexpectedly, C-terminal TRIM5αhu sequences mildly strengthened the late restriction activities of TRIM5αag and TRIM5αcy; while impaired late restriction activities were observed when the N-terminal region of TRIM5αrh was fused with C-terminal TRIM5αag or TRIM5αcy sequences (Fig. 6C). These results indicate that TRIM5α RBCC sequences are required for the late restriction, and C-terminal amino acid sequences can modulate the potency of the late restriction against HIV-1, adding further complexity to the mechanisms of TRIM5α-mediated late restriction. Further studies will determine the extent of inter-domain communication within the TRIM5α protein and the amino acid residues which are responsible for the negative regulation.

Proteins that have a conserved RING, b-box 1 and/or b-box 2 and coiled-coil domains are included in the superfamily of TRIM genes, and many TRIM proteins have been implicated to be interferon-responsive and a contributing factor in the defense against infectious agents [42], [51]. Located in the same genetic locus on chromosome 11p15, TRIM5, TRIM22, TRIM6 and TRIM34 were classified in the same clade [22]. Of the human TRIM proteins assessed in this study, TRIM21 and TRIM22 were efficiently incorporated into VLPs. A faint TRIM18 signal was also detected in the purified VLPs, suggesting weak interaction between human TRIM18 and HIV-1 Gag (Fig. 7A). In contrast to the efficient encapsidation of these proteins, only TRIM22 showed a modest late restriction activity against HIV-1 (Fig. 7B and C). These data suggest that encapsidation efficiency alone could not fully explain the differences in the late restriction activities of TRIM proteins. It is likely that additional determinant(s) controls the potency of the late restriction activity following initial binding of a TRIM protein with HIV-1 Gag.

In summary, we demonstrated the examples of TRIM protein-mediated late restriction activities and their potential to interact with viral proteins. Our data provide further insights into the complex host-pathogen interplay in TRIM protein-mediated late restriction.
